# Dental students’ perspective on learning activities regarding professionalism. A meta ethnographic review

**DOI:** 10.3389/fdmed.2025.1603222

**Published:** 2025-12-10

**Authors:** Christina Gummesson, Andreas Agouropoulos, Melanie Nasseripour

**Affiliations:** 1 Faculty of Odontology, Malmö University Malmö and Faculty of Medicine, Lund University, Lund, Sweden; 2 School of Dentistry, National and Kapodistrian University of Athens, Athens, Greece; 3 Faculty of Dentistry, Oral and Craniofacial Science, Centre for Oral, Clinical & Translational Sciences, King’s College London, London, United Kingdom

**Keywords:** meta-ethnographic analysis, professionalism, professional identity formation, oral health education, dental students

## Abstract

**Introduction:**

Present literature on professionalism usually focuses on individual parts of professional identity development and mostly, as these are seen and are evaluated by the dental educators. When looking at professionalism and in particular professional identity formation, It is extremely important to listen to the voice of the students regarding their thoughts, attitudes, beliefs and ideas about how to become ethical, compassionate, and culturally competent oral health professionals. The aim of this meta-ethnographic review was therefore to explore students’ perspective on learning activities regarding professionalism.

**Methods:**

A review of the literature was conducted following the PRISMA guidelines to identify qualitative studies on professionalism teaching, focusing on dental students, covering from 2010 up to May 2024. Critical appraisal of the articles was conducted by 2 researchers using the Joana Briggs Institute and the McGill Mixed Methods Appraisal Tools. We then applied the meta-ethnographic methodology defining our aim, and proceeding with in depth reading, relating and translating of first and second order point of views before drawing our own salient themes.

**Results:**

From the initial 6,995 studies, 11 were included in the analysis. The studies represented 6 different countries, 8 were of high and 3 of moderate quality. A conceptual preliminary matrix was developed based on the first reading and then used to compare quotations from the different studies. The overall themes in the conceptual model were phrased “Intentionality” and “Learning Context”. The analysis of the quotations from the informants (first order) and the researchers (second order) yielded five sub-themes: changed self-awareness, perseverance, safe learning environment, authenticity, and shifting perspective.

**Discussion:**

Dental educators should understand the students’ perspective and needs for professional identity formation and understand their own position as role models towards this direction. Dental education should be a safe learning environment where multiple stimuli provided together with reflection and exchange of thoughts guided by educators, enhance development of students’ professional behaviour.

## Introduction

Dental education refers to the structured process of teaching and training individuals to become competent dental professionals capable of diagnosing, treating, and preventing oral health issues. It encompasses a combination of theoretical knowledge, clinical skills, ethical principles, and professional behaviours, necessary for delivering high-quality patient care. An integral part of this education is the development of professional behaviours by the new dentist and this asset is fully recognized in the competencies for the new dentists by international education bodies (1).

Professionalism is a cornerstone of dental practice and is integral to the trust placed in dentists by their patients and the public. According to the ADEE definition in the first domain of competences for the graduating European dentist, professionalism is a commitment to a set of values, behaviours and relationships, which underpin the trust that the public holds in dental care professionals (2). The development of a set of attitudes, behaviours, and ethical principles that underpin clinical competence, is essential to delivering high-quality patient care and maintaining the integrity of the profession. Therefore, the importance of teaching professionalism in dental education has gained increasing attention in recent years, as it is recognized by all stakeholders (government, scientific and educational bodies, universities etc) the need to prepare students for the multifaceted demands of modern dental practice (3).

Dental schools bear a critical responsibility in fostering professionalism among students. However, teaching professionalism presents challenges (4). Students must learn to navigate complex ethical dilemmas, communicate effectively with diverse patient populations, and uphold the highest standards of integrity. Consequently, educators must adopt innovative and holistic strategies to integrate professionalism into the dental curriculum, ensuring that students are not only technically proficient but also morally grounded and socially responsive practitioners. In a recent review on the current state of professionalism in dental curricula, it was noted that there is a great variety in the pedagogical methods used and the contexts of the subject (5).

Professional behaviour is further challenged by the advancements in technology such as the use of social media and AI during dental education and later during professional life. Furthermore, another challenge in the pedagogy of professionalism in the medical fields is the generational gap between teachers and learners (6). It is extremely important to listen to the voice of the students regarding their thoughts, attitudes, beliefs and ideas about how to become ethical, compassionate, and culturally competent dental professionals. Several articles have tried to address different aspects of professionalism through qualitative approaches that deliver insight into the different topics (7–10). Dental education is demanding and stressful as students must learn while also developing their professional identity. Students identify the need to incorporate key concepts like effective role modeling and mentoring by the faculty within a safe learning environment (7), and also note barriers to approaching faculty (8). Moreover, concerns like gender differences (9) and racism (10) have been raised by dental students, affecting their wellbeing and resilience. Previous studies have identified institutional gaps most focus on specific segments, while a holist approach could be more beneficial for schools to provide better education regarding professionalism in dental curricula. Nevertheless, to our knowledge, there is no systematic review of such studies that could provide a more comprehensive view and identify key concepts that could assist dental educators and institutions to teach professionalism in a more effective and appropriate way. Therefore, the aim of this meta-ethnographic review was to explore students’ perspective on learning activities regarding professionalism.

## Methods

The literature search was initially conducted and is described in detail, in a scoping review on teaching professionalism in dental curricula (5). The systematic review of the literature was conducted following the Preferred Reporting Items for Systematic reviews and Meta-analysis (PRISMA) guidelines to identify recommendations/ evidence to be included in professionalism teaching for dental curriculum. The initial review included studies up to 2023 and it was repeated to include studies published up to May 2024. From the 35 articles of the initial review 9 fulfilled the inclusion criteria for this study, while two more were identified in the updated search. Critical appraisal of the 11 articles was conducted by 2 researchers (AA, MN) using the Joanna Briggs Institute tools (JBI) for qualitative research appraisal tool as well as the McGill Mixed Methods Appraisal Tool (MMAT) (11–14). The McGill MMAT tool is adresses the quality of mixed methods studies (appraisal of qualitative, quantitative and mixed methods components). Whereas JBI was used for purely qualitative study designs. It was established that consensus was reached when both appraisers agreed. Data extraction was performed by 3 different authors (AA, MN, CG) separately, in case of disagreement a consensus position was achieved after considering the analysis of the 3 authors. To perform the qualitative analysis in this study, we followed the steps described by Noblit et al. (15). These steps are: getting started by clarifying the aim of the study, deciding what is relevant, reading the studies, determining how the studies are related, translating the studies into one another, synthesizing translations expressing the synthesis.

### Literature search

**Figure 1:**
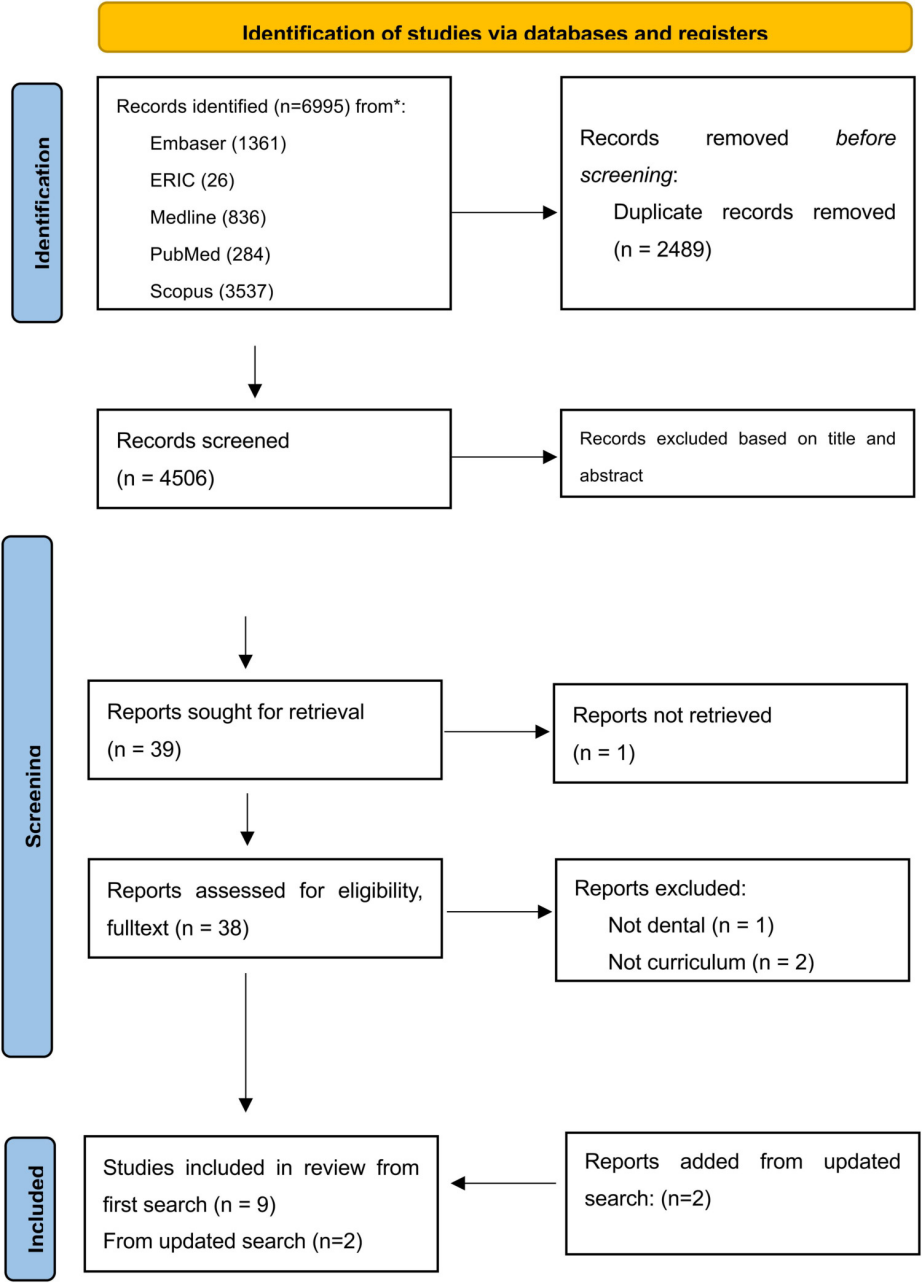
Flowchart of the section of the included articles in the present review. Source: Page MJ, et al. BMJ 2021;372:n71. doi: 10.1136/bmj.n71. This work is licensed under CC BY 4.0. To view a copy of this license, visit https://creativecommons.org/licenses/by/4.0/.

The search strategy was established using the following query: (dent* or odontology) AND [(professionalism) OR (ethics) OR (deontology)]. We included manuscripts Filters in English language and spanning 2010 through 2023. In the databases searched (Embase, ERIC, Medline, Pubmed, Scopus and Web of Science) From the initial 6,995 records, 9 articles met the inclusion criteria included and selected for full text analysis. The search was then updated June 2024 and 2 more studies were added, Figure 1.

### Selection criteria

Studies in English language, that employed qualitative or mixed-method studies including data (quotes) from dental students.

### Data analysis

Data were extracted from the studies to describe the study characteristics: author, country, year of publication, study aim, methods, study participants. The papers included were read and reread by the researchers several times. We decided to read the studies in a chronological order. To determine if or how the studies were related to another, the student quotes (first-order data) were coded to compare and contrast the findings and second-order data (what the study-authors expressed) was also included when we described a first draft of key themes and categories. This was repeated iteratively to form a translation of findings across the studies. Our synthesis of the translation is then expressed as a third-order interpretation.

### Research team and reflexivity

All three researchers are senior educators working in dental education with a diverse background:

CG is working with educational development and research, she is not a dentist but has a background within the health professions. AA has been involved in dental education of undergraduate and postgraduate students for more than 22 years. MN has over 20 years of experience in Undergraduate clinical education and a special interest in Ethics and Professionalism which has translated into educational research into reflective practice and metacognition. We are all 3 members of the steering group for the Association for Dental Educators Europe (ADEE) Community of Practice in Professionalism interested in the place of professionalism education within dental and oral health curricula and more specifically in learning and sharing about students’ experiences of curricula which influence their professionalism. We decided that all researchers should read all papers, to ensure a rich discussion and interpretation ground.

## Results

**Figure 2:**
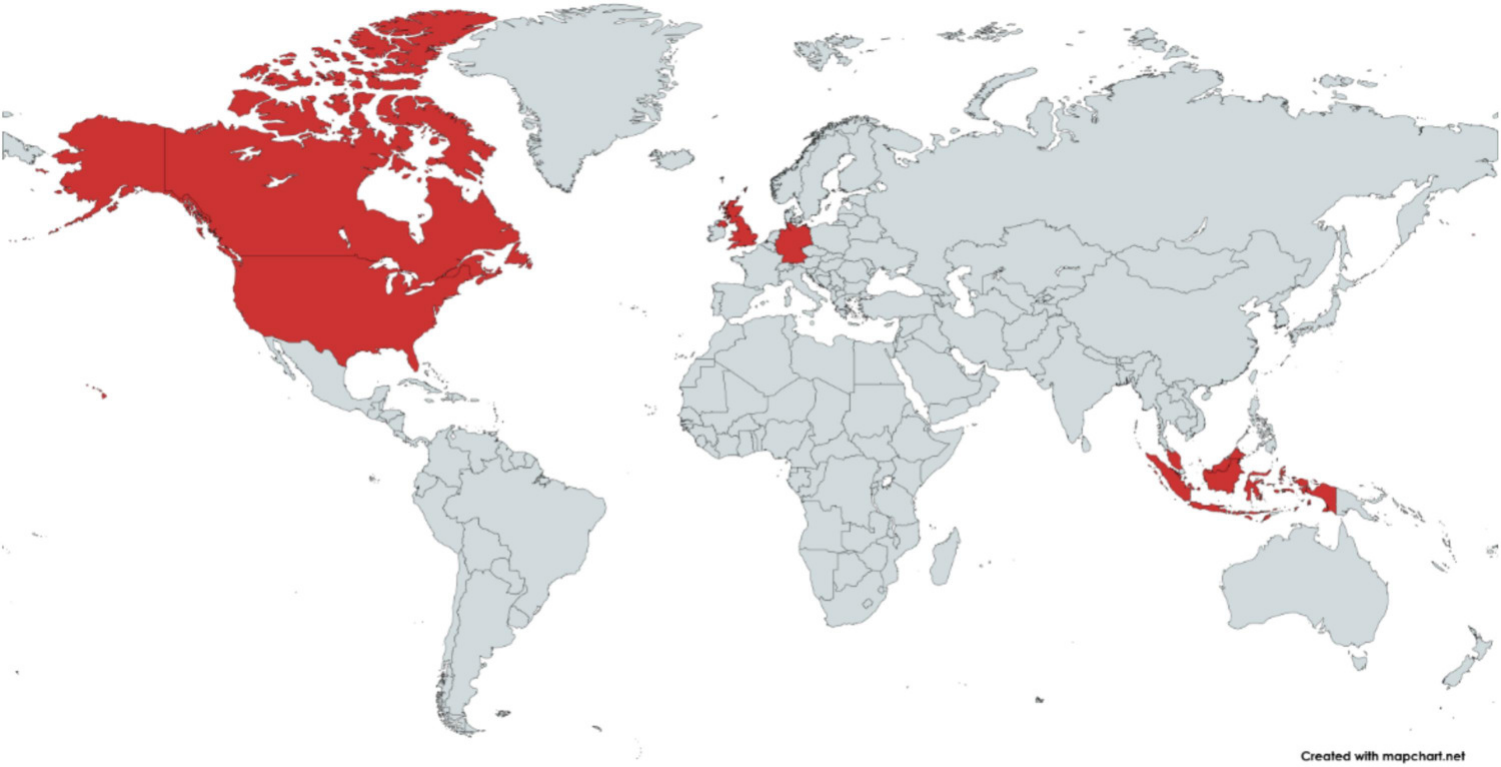
Graphic representation of county of origin of the included studies.

Eleven studies using a qualitative approach were included. The studies were conducted in the UK (10, 16–19), US (20, 21), Canada (7), Malaysia (22), Indonesia (23), and Germany (24), (Table 1 and Figure 2). All studies were rated to be of high (*n* = 8) or moderate (*n* = 3) quality (Table 1).

**Table 1:** Characteristics of the included studies and quality assessment.

Author and date	Country	Study design	Participants	Aim	Critical appraisal
Behar-Horenstein et al. 2015 (20)	USA	Qualitative	Dental students (undergraduate before 1st year)	“To explore how a service -learning experience affected a small group of dental students’ beliefs about cultural competence, professionalism, career development, desire to practice in a community service setting, and perceptions about access and disparities issues”	High^a^
Ranauta et al. 2018 (17)	UK	Qualitative	Dental students (undergraduate, years 1–5)	“To explore which learning experiences dental students perceived as influential in developing their understanding and enactment of professionalism. In turn, this could inform the development of dental curricula”	High^a^
Knott et al. 2018 (16)	UK	Mixed methods	Dental students (undergraduate, 1st year)	“To explore the views of the first-year graduate entry programme students at the University of Central Lancashire and their use of SMS together with their opinions on what they consider to be professional online behaviour”	Moderate^b^
Quick & Blue, 2019 (21)	USA	Mixed methods	Dental students (undergraduate, 1st year)	“To investigate the effectiveness of a project based on situated learning theory and using an Ignite format to support dental students’ learning and promote professionalism”	High^b^
Greviana et al. 2020 (23)	Indonesia	Qualitative	Dental students (undergraduate, years not mentioned)	“To explore the process of selecting and reflecting upon evidence of professionalism recorded in e-portfolios during undergraduate clinical dentistry training”	High^a^
Ahmad et al. 2020 (22)	Malaysia	Qualitative	Dental students (undergraduate, years 1–5)	“To investigate the effects of an extramural educational program involving people with disabilities on students’ professionalism and their perception of special care dentistry education”	Moderate^a^
Gormley et al. 2021 (18)	UK	Qualitative	Dental students (undergraduate, years 2–5)	“To explore the impact of digital professionalism awareness raising activities provided at one UK-based institution, using our “brown envelope” intervention”	High^a^
Dargue et al. 2021 (19)	UK	Qualitative	Dental students (undergraduate, years 3–5)	“To explore the undergraduate dental clinical students’ experiences and perspectives of paired working in the clinical learning environment”	High^a^
Ahmadifard 2022 (10)	UK	Qualitative	Dental students (undergraduate, years 1–5)	“To gain a deeper understanding of the perceptions and experiences of dental students or racism, discrimination, and inequality in a UK dental school. We also explored the impacts of racism on dental students and students’ responses to racism to identify any barriers that hinder effectively reporting or dealing with racial harassment and discrimination”	High^a^
Kwon et al. 2022 (7)	Canada	Qualitative	Dental students (undergraduate, 4th year)	“To investigate and analyze the relevant components of professional identity in dental students, and explore students’ concerns toward their professional identity formation”	Moderate^a^
Sebastian et al. 2023 (24)	Germany	Qualitative	Dental students (undergraduate, years 4–5)	“To determine how dental students experience their first oral and maxillofacial surgery internship in terms of their concept of professionalism and their perception of role models”	High^a^

a JBI appraisal tool.

b McGill MMAT tool.

A conceptual preliminary matrix was developed based on the first reading and then used to compare quotations from the different studies (Table 2). The overall themes in our conceptual model were phrased ‘Intentionality’ and ‘Learning Context’. The analysis of the quotations from the informants (first order) and the researchers (second order) yielded five sub-themes: changed self-awareness, perseverance, safe learning environment, authenticity, and shifting perspective (Table 2).

**Table 2:** The themes were developed based on the five sub-themes, here presented with examples from the interpretation and selected sample evidence from the papers.

Theme	Sub-theme	Selected Sample Evidence (First order information and Second order Constructs)	Third order interpretation
Intentionality	Changed self-awareness	“I have to learn to reserve my opinions and judgments in order to treat patients that I cannot understand” (20) “The actual physical act of treating a patient I think makes you step up, makes you act professional” (17) “I was just not that aware of, like, people having my information, and being able to access it as easily… as well as what was put in the envelope. So yes, I think that was really useful”. (18) “I learned that there is a lot to dentistry that we have to learn that doesn't necessarily have to do with teeth.” (21) “We want to learn more. But please don't impose clinical requirements. Otherwise, we will feel “insincere.” As if..we treat them as an object to fulfil our clinical requirements, rather than treating them as a human being.” (22)	Concrete learning activities can promote a broader understanding of the role and impact a dentist (student) may have through their behaviours.
	Perseverance	“While also considering himself a future practitioner, [Z] reported that he “would save a certain percentage of my income to a “special account,”” implying that he would want to help finance dental care for those who could not afford it.[Y] remarked that he would remember this experience and would work to solve these problems when he became a dentist, while [W] found himself feeling encouraged to consider a specialization in pediatric dentistry” (20) “I learned that it really is very important to speak up if you see something unethical happening. Although it may be hard, it is the right thing to do and is necessary for retaining your integrity. As a professional, this applies to the real world as well. You need to have the courage and the moral values to report any unethical behaviours or problems you see to maintain the reputation of the profession and to ensure the safety and well-being of the patients” (21)	Intention to take a long-term responsibility
		“Dental students were highly motivated to develop their professional role within clinical environments providing a range of dental care for diverse patient needs. Students recalled how positive and negative behaviours of colleagues influenced their developing sense of professionalism.” (17) “Well […] it had an effect on me in that I always used to think about whether I might want to go into surgery myself. But then I realized that it suits me better […] to work as a dentist and to still have the interpersonal relationships. And […] that it doesn't really suit me, this, let me say, cold […] work and somewhat more detached work, like I experienced there.” (24)	Intentional ways of achieving their goals
Learning context	Authenticity	“The best way of learning about professionalism is watching someone who's got vast experience and has been there and done it, and yes, copying” (17) “You can see certain tutors, not really acting professionally. Their attitudes, time management and all I used to think was I hope I'll never be like that”. (17) “I think they [University] do need to focus on the positives a bit more. Because I think it was all, ‘Don't show you ever socialise, don't show you ever go out, don't show this, don't show that.” (18) “It's good to learn how to work with other people, because that's what you will be doing for the rest of your life.” (19)	An authentic situation may be created by complex, messy learning activities requiring students to explore, problem solving and meaningful learning (as opposed to rote learning). However it is also important to note that every teacher needs to be aware of them being seen as role models all the time.
		“These situations occur because of stress, mob mentality, and more…I took away from the Ignite presentations an ability to detect and deal with these types of situations. The stress will always be there, but it is up to the individual to deal with the stress.” (21) “I find you learn a lot indirectly simply by being in an observational […] role as a student and often simply being present more or less passively […]. At first, you might often ask yourself what you're actually doing there because you can't do anything practical, you're not learning anything. Because I think we are used to doing a lot of practical things in our studies and learning by doing. But even dealing with things, whether it be with stress or patients, or absorbing any specialized information along the way. I think that contributed even more to the learning effect.” (24)	The role of emotions
	Safe learning environment	“Someone asked you a question… and it gets you thinking to answer the question, but that's only if you get someone who cares enough to ask, and to be engaged in the session” “The data also shed some light on the need to offer more educational support to this group of students without the assumption that ‘they know better.” (16) “..she kind of made it into a positive learning experience. She didn't punish you for anything. She always made herself available..” (7)	The need for a non-judgemental environment to explore the unknown
		“Respondents in this study were unfamiliar with the concept of self-reflection; therefore, they required more time and training to demonstrate good self-reflection skills. The longer use of a reflective e-portfolio may actually facilitate their need to practice to improve their self-reflection skills.” (23) “The most effective aspect of this project was the guided discussion that followed the presentations. Time to talk to one another and to the professors helped us gain more insight on how each of these topics affect us as dental students and what we can do when we might encounter similar situations”. (21)	Opportunities for reflection and exchange
	Shifting perspectives	“To understand patients unlike myself, I learned to take time to sit down with them and listen to them on their level to truly understand their story and experiences in life.” (16) “We learned that allowing each team member to contribute is key to a diverse and well-rounded finished product. However, it is important to realize that each team member's contribution will be different.” (21) “What helps a lot while writing reflections is when I get detailed feedback from the supervisor while treating patients, describing what was good or bad and why. I use the feedback as a starting point for making self-reflections.” (23) “…you still have to put the patient's interest first so does it really matter that the patient likes or doesn't like you? But you have a duty to care and if that means putting your patient out of pain and that's what you do, you put a patient out of pain”. (10)	Exposure and reflection enables the understanding of other people

### Line-of-argument synthesis

#### Intentionality—self-awareness and perseverance

*Intentionality* was shown as *changed self-awareness* and *perseverance* where the students expressed different examples of how they after learning activities intentionally adjusted their present behaviours and were triggered to plan for how they could improve, when they realized that they now represented a profession. Learning activities where theory and practice were integrated, and expectations were clarified seemed to be important to promote a broader understanding of their current and future role and impact. Students expressed new intentional behaviours, based on gained knowledge and reflections about professionalism (10, 18, 23, 25) and clinical experiences (7, 17, 19–22, 24). Changes in how students became intentional in how they acted to be professional, were noted in various ways across the studies, but the students intentionally seemed to develop during the education, supported by the learning activities.

The self-awareness seemed to develop by the students becoming aware of disparities, realizing their responsibility, how they could have great impact on other peoples’ lives in their future role (20, 22). A similar self-awareness was expressed into consequences of their social media activities (16, 18) and how they through these activities intentionally could impact professional boundaries and their future patient relations in positive or negative ways. Students also expressed awareness of the consequences of showing emotions such as stress could be perceived if it was shown to patients (21).

Students expressed a desire to develop self-efficacy to become competent dentists (7). Further, in Knott et al. (16) it was concluded that education in professionalism needs to be repeated at different occasions during the education. Ranauta et al. (17) described the need for a theoretical foundation, in combination with perseverance and individual responsibility, was needed to move from “knowing” to “acting” to “being” professional. The students also expressed the awareness of their future ability to help others being a source for perseverance when they could relate to good role models; senior students (19), teachers (17) and clinical supervisors (24). However, role models can be both positive and negative, and students became aware of the power relation challenges within the university (10). Our third order interpretation was that concrete learning activities integrating theory and practice could enable a broader understanding of the role and impact a dentist may have and promote motivation to change behaviours.

#### Learning context—authenticity, safe learning environment, shifting perspectives

Students across the studies expressed the importance of the learning context to develop professionalism. The *Learning Context* included aspects of *authenticity*, where interaction with different persons (teachers, supervisors as role models, peers, patients) throughout the education was seen to have large impact on the students’ ways of thinking about their behaviours and communication. To develop, students expressed the need of *safe learning environments* where one would dare to ask questions, reflect and exchange thoughts guided by teachers. An authentic and safe learning environment in clinical settings with concrete experiences, such as challenging patient encounters (20, 22), powerful emotions (17), students for peer learning and benchmarking (19) and teachers as role models (17) could promote professionalism development. Though authenticity is desired, if not set as mandatory it can sometimes be avoided. Greviana et al. (23) concluded that their results about the use of reflections suggested that most trainees only documented successful and completed activities.

As part of the learning context, it also seemed to have large impact when students were put in positions where their own perspective was challenged by being exposed to others’ perspective. It seemed that *shifting perspective* during learning activities enabled students to develop tools to build professional relationships with team members and patients. It seemed to be valuable to be exposed to shifting perspectives to explore how other people are experiencing their own lives and roles (20, 22) to shift to prioritize the patient's needs in front of your own (22, 23) or to explore how other people are experiences the student behaviours e.g on social media (16, 18) or guided discussion (21).

## Discussion

In this systematic review of qualitative data on dental student's perspectives two main themes (intentionality and learning context) and five sub-themes (changed self-awareness, perseverance, safe learning environment, authenticity, shifting perspective) were described.

Intentionality, meaning that students behave and communicate actively and consciously (with specific intentions) to reach their goals, is not usually described as a facet of professionalism education. However, through reading across the studies, we noted that students expressed, after the learning activities, intentionally changing their behaviours as part of becoming a professional. Intentionality may be defined in various ways, as it is often looked at more from a philosophical perspective. An intention can be strictly defined as “something that you want and plan to do” (26).

We defined one subtheme as changed self-awareness. Interestingly the Harvard Business school identifies two broad categories of self-awareness: “Internal self-awareness, which relates to how clearly you see your own values, strengths, weaknesses and its impact on yourself and others, and External awareness, which relates to understanding how others view us in terms of our values, strengths, weaknesses and impact” (27). The key word here is the change in self-awareness which we could see as coming from the realization of the external awareness and effect on the internal self-awareness.

We argue here that this change in self-awareness links to the process of professional identity formation often mentioned in health education. Conceptually, we are looking at an individual's existing identity and an aspirational identity which is intentionally pursued as a goal and stems from the interactions with patients, colleagues/peers, educators and the profession. It is a life-long individual, psychosocially grounded, contextual journey “of deconstruction and reconstruction depending on how the person experiences, and thus responds to, events”.

Through the lens of professional identity formation (28) we can better appreciate the notion of perseverance which we highlighted as this journey is one of successes and turbulations. It is a journey which tests the students resolve and requires a support system derived from a community of practice which can be drawn from self, peers and teachers. Perseverance is a “continued effort to do or achieve something, even when this is difficult or takes a long time”. This could be seen as a broader concept than resilience, which looks more at the “the ability to be happy, successful, etc. again after something difficult or bad has happened” (29). The students’ responses to and lived experiences of this journey may shape their professional identity.

This brings into focus the theme of the learning context. This is here seen in a broad sense, not only during scheduled learning activities, but taking the learning environment into consideration, such as teachers’ behaviours in student and patient encounters. The students are across the studies describing the context similar to what could be expected in a professional community of practice (30). In a community of practice, participants may be in the core or peripheral, but all learning as part of the process of social participation in a group with a shared endeavor. During their education, the students are peripheral participants, recognizing the teachers and clinical supervisors as the community of dentists they are striving to belong to and seeing as role models. Students also perceived community practice as contributing to professional identity formation. To be recognized as being part of the professional community could be interpreted as a responsible action. Community of practices galvanize knowledge sharing, learning, and change in free-flowing, creative ways that foster new approaches to problems. We also here are using the term, emphasizing the idea of a community supporting the learner in their journey to professional identity formation. This is where peer learning comes into play with students’ recalling how both positive and negative behaviours influenced their perception.

We mentioned the psychosocial aspect of the professional identity formation, and this brings in the subtheme of safe learning environment. It is essential to appreciate the psychological aspect and that students feel safe to make mistakes and ask for help. A “safe learning environment”, which should come with guidance, time to practice, explore and ask questions, seems important throughout the education. Students highlighted that they appreciated when a teacher made the situation into a positive learning experience. It is well recognized that a learning environment where students feel safe, with a curious and creative atmosphere, is preferable for effective learning and this was confirmed in the present study. The safe learning environment though, needs to remain real, and its authenticity is key to bring value to the event for students.

Authenticity, could be defined as being complex, requiring exploration, problem solving and meaningful learning [as opposed to rote learning (31)]. In the oral health curricula, authenticity is often related to what the clinical environment or scenario can bring, but also often there is al contrast between school environment and practice with outside practice. The concern is to prepare students for an authentic practice not an ideal and unachievable one. For most teachers the clinical setting is probably recognized as authentic, but learning activities that are seen as authentic can be developed in various environments. It is more about how the challenges are presented to the students.

What also comes with the learning context is different perspectives. We specifically use the term “shifting perspective”, meaning that somebody is challenged to understand how other people are thinking and feeling and using that to guide his or her behaviours. Being able to understand different perspectives is essential in healthcare, since there is the patient's perspective, the clinician's perspective, the team or other staff's perspective, and also general public perspectives. We do not practice oral health care in isolation.

After conducting this study, we believe that intentionality expressed through changed self-awareness and perseverance should be recognized as part of the outcome of professionalism education. Increased self-awareness, (i.e., understanding of one's own emotions, thoughts and values and how they influence behaviours) and perseverance, (i.e to intentionally strive for goals that are not easily or fast achievable and not giving up) are traits that once mastered may not be forgotten. One could argue that intentionality seems to be a threshold concept for professionalism (32). In a study from Canada where faculty members were interviewed about threshold concepts for dental education, the four concepts described were “dealing with the whole patient”, “accountability”, “that you may not know everything” and “problem solving and adapting during practice” (33). The concepts are holistic, and we believe that once the concepts are mastered, they align with what we here described as intentionality in professionalism. It would be beneficial for the students if the professional identity formation was explicit during education and the concept of community of practice could be an enabler. The identity formation is an important part of professionalism, and we believe that also being explicit to all teachers about their impact as role models should not be underestimated.

It is well recognized that a learning environment where students feel safe with a curious and creative atmosphere is preferable for effective learning. This was confirmed in this study. A “safe learning environment” with guidance, time to practice, explore and ask questions seems important throughout the education. The third sub-theme was “Shifting perspective”, meaning that you are challenged to understand how other people are thinking and feeling and using that to guide your behaviours. Based on our findings here, we recommend that contextual aspects of authenticity, safe learning environment and shifting perspective should guide the design of learning activities to foster professionalism.

Developing professional identity is inherently constructivist, identity is not taught, rather constructed through personal and social experience, in line with socio-constructivist learning theory, here within the context of dental education. Students through learning activities, experience, social interaction and reflection construct knowledge actively and become dental professionals. Therefore, adopting a holist approach can benefit dental curricula to provide better education regarding professionalism. To our knowledge this is the first meta-ethnographic review that focuses exclusively on dental students’ perspectives regarding professionalism, and adds this holistic approach providing important insights into the topic. The inclusion of students in different years in their studies and of different aspects of professionalism also adds to the value of this review since professional identity is built over time and on different aspects. There is a representation from different parts of the world which is important due to the cultural aspect of professionalism and most of the studies were of high quality increasing the validity of the results. Furthermore, the authors have different backgrounds in dental education and working experience which adds to the approach of the review. Nevertheless, it should be considered, as in any qualitative research, that these are the author's interpretations. We have strived for trustworthiness by reading and rereading the texts, regular meeting to discuss and reflect about the interpretation and meaning, relating our finding to previous research (34). Some parts of the world are not represented in this review while most studies originate from western culture countries which may limit the applicability of the results in other cultures.

Development of professional identity is probably the most challenging aspect of dental education. Professionalism is complex and difficult to evaluate competency for the dental graduate. It is dynamic, multi-faceted and context dependent with several stakeholders that need to be aligned for successful teaching (35). Nevertheless, dental educators are the key for training and development of this competency and should understand the students’ perspective and needs for professional identity formation. Teachers should not underestimate their position as role models not only in the clinical setting but in every educational activity and interaction with the students. Dental education should be a safe learning environment where multiple stimuli provided together with reflection and exchange of thoughts, guided by educators, enhance development of students’ professional behaviours. Listening to the students’ voice is extremely important, especially because of the generation differences that lead to different perspectives and demands from both sides. A shared understating is necessary, and the present review provides an insight on the students’ side that could lead to better communication between parts. Furthermore, the identification of the presented themes could assist in a more organized approach in curriculum development on teaching professionalism in dental institutions. To foster this approach interactive learning activities such as structured reflective practice, simulation-based learning and more holistic evaluation approach e.g., 360-degree evaluation, could improve professional identity formation and evaluation of professional skills. Dental institutions should also promote and assist faculty development, to enhance the educator's role as mentors and role models.

## Conclusions

This meta-ethnographic review of the available literature provides a holistic approach on students’ professional identity formation as seen and perceived by them. Two main and five sub-themes were identified: intentionality (self-awareness and perseverance) and learning context (authenticity, safe learning environment, shifting perspectives). We found that when students realize that they represent the dental profession, they intentionally adjust their behaviours by becoming self-aware of the different aspects of dentistry. Perseverance and individual responsibility were identified as prerequisites to move from theory to practice and formation of a professional identity. Moreover, development of professional identity can be achieved better in a safe learning environment where students can interact with others and therefore develop appropriate behaviours while shifting their perspective during learning activities enabling them to develop tools and to build professional relationships with others. Based on the above, enhancement of the educator's role as mentors and role models through faculty development and adoption of interactive learning activities and a holistic evaluation approach can improve professional identity formation and evaluation in the dental curricula.

## Data Availability

The raw data supporting the conclusions of this article will be made available by the authors, without undue reservation.

## References

[1] Cowpe J Plasschaert A Harzer W Vinkka-Puhakka H Walmsley AD. Profile and competences for the graduating European dentist - update 2009. Eur J Dent Educ. (2010) 14(4):193–202. 10.1111/j.1600-0579.2009.00609.x 20946246

[2] Field J Vital S Dixon J Murphy D Davies J. The graduating European dentist curriculum framework: a 7-year review. Eur J Dent Educ. (2025) 29(1):155–61. 39563643 10.1111/eje.13058 PMC11729984

[3] Cunningham IM Gormley M Neville P. Contemporary dental student professionalism: moving towards a macro-level perspective. Br Dent J. (2024) 236(8):631–6. 10.1038/s41415-024-7297-8 38671122 PMC11052704

[4] Nguyen TM Jones D Ngo KL Hayes MJ. Developing professionalism in dentistry: a systematic review. MedEdPublish. (2017) 6:85. 10.15694/mep.2017.000085 38406420 PMC10885255

[5] Nasseripour M Agouropoulos A Van Harten MT Correia M Sabri N Rollman A. Current state of professionalism curriculum in oral health education. Eur J Dent Educ. (2025) 29(1):92–103. 10.1111/eje.13048 39501916 PMC11730743

[6] Schnapp BH Cloyd T Hartman ND Moadel T Santen SA Gottlieb M. Avocado toasted: mythbusting “millennials,” “generation Z,” and generational theory. AEM Educ Train. (2022) 6(3):e10757. 10.1002/aet2.10757 35664707 PMC9134576

[7] Kwon JH Shuler CF von Bergmann H. Professional identity formation: the key contributors and dental students’ concerns. J Dent Educ. (2022) 86(3):288–97. 10.1002/jdd.12810 34697792

[8] Chan CCK Fok EHW Botelho MG. A qualitative analysis of Students’ perceptions and experiences of stressors and well-being in dentistry. Eur J Dent Educ. (2025) 29(1):195–210. 10.1111/eje.13062 39676268

[9] da Graça Kfouri M Moysés ST Gabardo MCL Moysés SJ. Gender differences in dental students’ professional expectations and attitudes: a qualitative study. Br Dent J. (2017) 223(6):441–5. 10.1038/sj.bdj.2017.810 28937117

[10] Ahmadifard A Forouhi S Waterhouse P Muirhead V. A student-led qualitative study to explore dental undergraduates’ understanding, experiences, and responses to racism in a dental school. J Public Health Dent. (2022) 82(Suppl 1):36–45. 10.1111/jphd.12514 35726468 PMC9328358

[11] Lockwood C Munn Z Porritt K. Qualitative research synthesis: methodological guidance for systematic reviewers utilizing meta-aggregation. Int J Evid Based Healthc. (2015) 13(3):179–87. 10.1097/XEB.0000000000000062 26262565

[12] Holden ACL. Incorporating humanities in dental education is essential, but seldom routine. J Evid Based Dent Pract. (2020) 20(2):101442. 10.1016/j.jebdp.2020.101442 32473810

[13] Aromataris E Fernandez R Godfrey CM Holly C Khalil H Tungpunkom P. Summarizing systematic reviews: methodological development, conduct and reporting of an umbrella review approach. Int J Evid Based Healthc. (2015) 13(3):132–40. 10.1097/XEB.0000000000000055 26360830

[14] Pluye P Hong QN. Combining the power of stories and the power of numbers: mixed methods research and mixed studies reviews. Annu Rev Public Health. (2014) 35:29–45. 10.1146/annurev-publhealth-032013-182440 24188053

[15] Noblit GW Hare RD. Meta-Ethnography: Synthesizing Qualitative Studies. Beverly Hills, CA: SAGE Publications (1988). 10.4135/9781412985000.n2

[16] Knott PN Wassif HS. Older and wiser? First year BDS graduate entry students and their views on using social media and professional practice. Br Dent J. (2018) 225(5):437–40. 10.1038/sj.bdj.2018.745 30168814

[17] Ranauta A Freeth D Davenport E. Developing understanding and enactment of professionalism: undergraduate dental students’ perceptions of influential experiences in this process. Br Dent J. (2018) 225:662–6. 10.1038/sj.bdj.2018.813 30287964

[18] Gormley M Collins L Schofield S Neville P. Exploring the impact of digital professionalism awareness training on dental undergraduate students. Eur J Dent Educ. (2021) 25(2):271–81. 10.1111/eje.12601 32949078

[19] Dargue A Richards C Fowler E. An exploration of the impact of working in pairs on the dental clinical learning environment: students’ views. Eur J Dent Educ. (2023) 27(1):87–100. 10.1111/eje.12780 35100467 PMC10078664

[20] Behar-Horenstein LS Feng X Roberts KW Gibbs M Catalanotto FA Hudson-Vassell CM. Developing dental Students’ awareness of health care disparities and desire to serve vulnerable populations through service-learning. J Dent Educ. (2015) 79(10):1189–200. 10.1002/j.0022-0337.2015.79.10.tb06012.x 26427778

[21] Quick KK Blue CM. Using situated learning theory to build an interactive learning environment to foster dental Students’ professionalism: an ignite project. J Dent Educ. (2019) 83(3):334–41. 10.21815/JDE.019.030 30692193

[22] Ahmad MS Mokhtar IW Khan NLA. Extramural oral health educational program involving individuals with disabilities: impact on dental Students’ professionalism. J Int Soc Prev Community Dent. (2020) 10(3):323–8. 10.4103/jispcd.JISPCD_74_20 32802779 PMC7402249

[23] Greviana N Mustika R Soemantri D. Development of e-portfolio in undergraduate clinical dentistry: how trainees select and reflect on evidence. Eur J Dent Educ. (2020) 24(2):320–7. 10.1111/eje.12502 31981383

[24] Sebastian T Prade A Keis O Schramm A Öchsner W. Student experiences of professionalism and role models in an oral and maxillofacial surgery internship: a qualitative study. Eur J Dent Educ. (2023) 27(4):849–58. 10.1111/eje.12875 36458891

[25] Arbaugh JB Cleveland-Innes M Diaz SR Garrison DR Ice P Richardson JC Developing a community of inquiry instrument: testing a measure of the community of inquiry framework using a multi-institutional sample. The Internet and Higher Education. (2008) 11(3):133–6. 10.1016/j.iheduc.2008.06.003

[26] Stanford Encyclopedia of Philosophy. Stanford, CA: Department of Philosophy, Stanford University. (2009). Available online at: https://plato.stanford.edu/entries/intention/ (Accessed December 01, 2025).

[27] Eurich T. Harvard Business school review Emotional Intelligence What Self-Awareness Really Is (and How to Cultivate It) (2018). Available online at: https://hbr.org/2018/01/what-self-awareness-really-is-and-how-to-cultivate-it (Accessed December 01, 2025).

[28] Sarraf-Yazdi S Teo YN How AEH Teo YH Goh S Kow CS A scoping review of professional identity formation in undergraduate medical education. J Gen Intern Med. (2021) 36(11):3511–21. 10.1007/s11606-021-07024-9 34406582 PMC8606368

[29] Cambridge Dictionary. Available online at: https://dictionary.cambridge.org/dictionary/english/resilience (AccessedJanuary 25, 2025).

[30] Wenger E. Communities of Practice: Learning, Meaning, and Identity. Cambridge: Cambridge University Press (1998).

[31] Mayer RE. Rote versus meaningful learning. Theory Pract. (2002) 41(4):226–32. 10.1207/s15430421tip4104_4

[32] Cousin G. An introduction to threshold concepts. Planet. (2006) 17(1):4–5. 10.11120/plan.2006.00170004

[33] Green J Rasmussen K. Becoming a dentist: faculty perceptions of student experiences with threshold concepts in a Canadian dental program. Can Med Educ J. (2018) 9(4):e102–e10. 10.36834/cmej.43351 30498548 PMC6260516

[34] Korstjens I Moser A. Series: practical guidance to qualitative research. Part 4: trustworthiness and publishing. Eur J Gen Pract. (2018) 24(1):120–4. 10.1080/13814788.2017.1375092 29202616 PMC8816392

[35] Hanks S Ranauta A Johnson I Bateman H Nasseripour M Neville P. Professionalism and dental education: in search of a shared understanding. Br Dent J. (2022) 232(7):470–4. 10.1038/s41415-022-4094-0 35396431

